# Gender Gap in Unhealthy Life Expectancy: The Role of Education Among Adults Aged 45+

**DOI:** 10.3389/ijph.2022.1604946

**Published:** 2022-08-24

**Authors:** Aïda Solé-Auró, Pilar Zueras, Mariona Lozano, Elisenda Rentería

**Affiliations:** ^1^ Department of Political and Social Sciences, Pompeu Fabra University, Barcelona, Spain; ^2^ Center for Demographic Studies, Autonomous University of Barcelona, Barcelona, Spain; ^3^ Institute for Social and Economic Research, Colchester, United Kingdom

**Keywords:** population health, gender paradox, disablement process, education gradient, Spain

## Abstract

**Objectives:** This paper examines the gender gap in unhealthy life expectancy across education levels and age in Spain to understand the extent to which the gender paradox exists over education and across ages.

**Methods:** Death registrations and vital status were taken from the Spanish Statistical Office, while the three health measures (chronic conditions, bad-self rated health and cognitive impairment) from the 2019 European Health Interview Survey. We used Sullivan’s method to compute unhealthy life expectancy by education level. We computed the gender and the education ratios of the proportion of unhealthy life years in each health measure by education and age.

**Results:** At almost all ages and all education levels, women significantly lived longer but in poorer health than men. Marked gender differences are seen across most age-groups, particularly among the low educated. We detected greater health inequalities by education level for women (confirming the gender paradox) and a health gradient due to aging and across the health measures charting the disablement process.

**Conclusion:** The new education distribution might improve the unhealthy life expectancy and might reduce the gender gap in the number of years spent in poor health.

## Introduction

Women’s life expectancy is higher than men’s at all ages in all countries of the world. Yet, while it is universally observed that male mortality exceeds that of females, studies examining gender differentials in morbidity generally find that women are in poorer health [1]; however, the morbidity difference is less apparent when studying gender inequalities in unhealthy life expectancy (UHLE) and the gender paradox (women living longer but unhealthier) might not be universal. Three general groups can be used to clarify the sex differences explanations in (un)healthy expectancies: biological factors, lifestyle behaviors and social profiles. For instance, variations in the gender health gap exist by age, time, country context [2, 3], and when using different health indicators [4]. Differences are also observed by education levels. In fact, education might contribute to the reduction of the gender paradox by acting in the less favored groups [5]. Overall, women present worse health conditions than men [6] as well as more intensive health care utilization [7]. Potential gender health differences in the use of different types of health measures (self-reported or diagnosed) should be considered. Researchers have debated on whether gender differences in reporting general health status and health conditions can bias non-objective health indicators and increase gender health differentials, although the general conclusion is that they are unlikely to be the main explanation [4].

This work examines gender differences in UHLE based on diagnosed and self-reported health indicators by education level in Spain. Disparities in health measures by sex and education over the life course confirm the relevance of this approach.

We consider education as a social determinant of health. In general, those with higher levels of education present longer life expectancies, lower mortality rates, and lower burdens of disease [8–10]. Therefore, higher-educated people are more likely to have better access to the knowledge of healthy lifestyles (i.e. higher quality food) and, at the same time, education reflects the capacity to have access to better health care. It is well documented that European women have experienced a large expansion of their educational attainment during the second half of the 20th century. However, this achievement does not seem to be reflected in a reduction of gender health inequalities, suggesting that the relationship between education and health differs by gender. For instance, absolute health inequalities by education level are more evident among women [11]. In addition, women with low education levels are the ones in the poorest health [10], and Spain is no exception [12–15].

Socioeconomic differences in mortality levels in Europe have historically been lowest in Spain [16, 17], yet they are among the highest when considering morbidity measures [13, 17, 18]. Specifically, Solé-Auró et al. [14] observed that in 2012 older Spanish adults presented a marked education gradient in healthy life expectancies. This gradient was larger for women in terms of the expected number of years living in poor health, despite their advantage in terms of life expectancy for all levels of education. Similarly, Blanes and Trias-Llimós [15] found a greater disadvantage of healthy life expectancy for women at age 30 in 2019.

Overall, our study aims to examine in depth female-male differences in UHLE by education status in Spain for middle-aged and older people. Interestingly, we used three health indicators that allow us to provide a more complete view of the appearance of several health outcomes while aging. This is of considerable relevance to the aging process and ties in closely with the study reported here as we also seek to highlight the need for a full understanding of health deterioration over the life-course by measures that generally follow on consecutively over the process. Thus, we studied the variation of the gender gap in health expectancy using three health measures and two levels of education in an effort to identify the main inequalities in the age-range considered.

## Methods

### Study Population

We drew on three data sources provided by the Spanish Statistical Office (Instituto Nacional de Estadística, INE) for individuals aged 45+. First, mortality data come from the 2019 Spanish National Registry of deaths by sex, age group, and level of educational attainment. We chose to use the 2019 figures because it is the year previous to the COVID-19 pandemic [19]. Second, the 2019 Spanish population by ten-year age groups and gender was obtained from the INE records for 45 to 85+ year-olds. We have not used five-year age groups as they do not guarantee a sufficient sample size by gender and education level. Finally, we calculated the prevalence of each of our three health indicators by ten-year age groups using microdata from the 2019 European Health Interview Survey in Spain (EHIS), a cross-sectional survey representative of the non-institutionalized population.

### Variables

We used three health status indicators to compute gender differences in UHLE based on the EHIS questionnaire. The importance of studying these indicators is key for understanding the appearance and the duration in life of different health measures while aging. First, we used self-perceived health over the previous 12 months, the original question being: “How is your health in general? Is it very good, good, regular, bad or very bad?“. We grouped these five responses into two categories: good (very good and good) (coded as 0) and poor health (regular, bad, and very bad) (coded as 1). Second, we analyzed the presence or absence of chronic conditions and risk factors. In line with Zueras and Renteria’s [20] study of trends in healthy life expectancy in Spain, we used an indicator that includes the following conditions: cancer, stroke, myocardial infarction, heart disease, hypertension, diabetes and high cholesterol, chronic low back and neck pain, asthma and chronic obstructive pulmonary disease. We considered individuals as presenting the health condition when they answered affirmatively to all three questions: “Have you ever suffered from ‘this specific health condition?”, “Have you had it in the last 12 months?” and “Has a doctor told you that you have it?”. Our last health measure concerns cognitive impairment for which respondents were asked “Do you have difficulties remembering or concentrating?”. We dichotomized the four possible answers into two categories: cognitively impaired (coded as 1) for those declaring themselves as having “some difficulty,” “a lot of difficulty” or “cannot do at all” and not cognitively impaired (coded as 0) for those reporting no difficulties.

Here, education serves as our indicator of socioeconomic status on the grounds that, first, it is more likely to remain constant during an adult lifespan and is easily measured for all individuals, even the economically inactive; second, it has been relatively well reported in questionnaires and survey data; and, third, the likelihood of reverse causation between education and health at older ages is lower than that between income/occupation and health [21]. We classified our respondents into two groups in line with the International Standard Classification of Education: First, “Low Education,” corresponding to those with compulsory education or lower, which includes the first stage of secondary education, primary credentials, as well as illiterate individuals (codes 1 to 4 in the original data source); and, second, “High Education,” corresponding to those with upper secondary, higher secondary credentials, vocational training, and university degrees at all levels, BA graduates, masters’ and PhDs (codes 5–9) (INE). To compute the life tables, we used data from EHIS to calculate the weighted proportion of the presence of the three morbidity health measures considered, within the two education levels and ten-year age groups, and for men and women separately.

### Statistical Analyses

First, we estimated life expectancy by each level of education and gender for the year 2019. As our abridged life table terminates in a large group at ages 85+, we calculated life expectancy at age 45 by computing the average number of years lived over this age. We chose 45 as it is safe to assume that for the majority of the population their education level will not change and, moreover, the question concerning cognitive impairment was only put to individuals of this age and above. Death registries presented missing data on the level of education in 2% of the cases that were distributed proportionally by the education levels in each age group. When comparing our estimated life expectancy by level of education with the ones published by INE [19] we found a small difference, that we attribute to different methods of imputing missing data.

We used Sullivan’s method to estimate Spanish UHLE [22, 23]. This is a prevalence-based method for dividing life-table years lived in an age interval into healthy and unhealthy years, based on the prevalence of each health measure in each age group. In this way, health expectancies are similar in their interpretation to total life expectancy, as they indicate the expected years lived in a state of good or bad health. The 2019 prevalence of the three health measures (EHIS) by education level and ten-year age groups are, therefore, extrapolated to the Spanish population data in the life table to estimate the expected life years spent in good or bad health. We estimated the unhealthy expectancies by 10-year age groups (45–54, 55 to 64, 65 to 74, 75 to 84, and 85+) and their corresponding 95% confidence interval [24], given the importance of accounting for uncertainty when reporting and interpreting healthy life expectancy estimates [25]. First, we presented the descriptive statistics of our health measures by level of education of men and women. Then we computed the gender ratios (male/female) of the proportion of UHLE (as a relative measure) in each state of each health measure and for the two education levels described to examine the magnitude of the significant differences observed in UHLE estimates. We also examined the education ratios (high/low) of the proportion of UHLE for men and women. Ratios above one indicate that men (or the highly educated) spend a larger proportion of their remaining life in a certain health state, whereas ratios below one indicate that women (or the poorly educated) spend a larger proportion in that health state.

## Results

**FIGURE 1:**
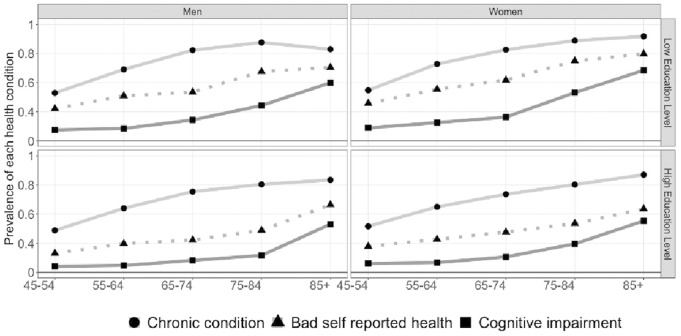
Descriptive Statistics: Prevalence of poor health condition for men and women by education level for ten-year age groups. Individuals aged 45+ (Source: European Health Interview Survey (EHIS), Spanish Statistical Office (INE), Spain, 2019).

Descriptive findings are reported in Table 1 and Figure 1. Table 1 presents the sample characteristics for the entire Spanish population aged 45+ by gender. The sample includes more women than men. The overall mean age is around 61.6 for men and 63.2 for women. Education levels differ slightly between genders. In general, men present higher levels of high education than women (69% vs. 63%, respectively). The prevalence of being in bad health for each health indicator is also higher for women compared to men. The statistical hypothesis test confirm that these gender differences are significant.

**TABLE 1:** Sample Characteristics: Sample size, mean age and standard deviation (sd), and weighted percentage of the education and health variables for Spain for men and women, Age 45+ (Source: European Health Interview Survey (EHIS), Spanish Statistical Office (INE), Spain, 2019).

	Men	Women
Sample Size (N)	6,899	8,111
Mean Age	61.6	63.2
SD Age	11.89	12.84
Low Education (in %)	31	37
High Education (in %)	69	63
Health variables (in %)		
Chronic conditions	57	61
Bad Self-rated health	30	39
Cognitive Impairment	12	18

Source: European Health Interview Survey (EHIS), Spanish Statistical Office (INE), 2019.

Figure 1 shows the prevalence of poor health among those aged 45 to 85+, for low and high education levels. Among those with low education, women report poorer health than men on all three-health indicators and across all ages. Gender differences in prevalence rates are less evident among those with high levels of education.

Table 2 depicts life and UHLE for men and women by level of education and age groups. Not only in all education groups but also at all ages, women can expect to live longer than men. Not surprisingly, there is a gradient in all health measures with aging, but we also detect a gradient across the health measures used. Thus, individuals spend more years with chronic conditions followed by years lived with bad self-perceived health, and finally, people spend less years with cognitive impairments at younger ages, as these only become substantially prevalent at older ages. For example, a man with low education level aged 45 years old is expected to live 35.2 years, and from those, 21 years will be lived with a chronic disease, 13.2 years with bad self-reported health, and 5.2 years with cognitive impairments. This health gradient pattern is consistent across age, education groups and genders.

**TABLE 2:** Life expectancy and unhealthy life expectancy (in years) for both men and women and for low and high levels of education by ten-year age groups (Spain, 2019).

	Life Expectancy				Years with health problems (UHLE)											
					Chronic condition				Bad self-reported health				Cognitive impairment			
	Men		Women		Men		Women		Men		Women		Men		Women	
	Years	CI	Years	CI	Years	CI	Years	CI	Years	CI	Years	CI	Years	CI	Years	CI
Primary or less																
45–54	35.24	(35.22–35.26)	40.8	(40.79–40.82)	20.96	(20.5–21.4)	24.73	(24.3–25.2)	13.2	(12.9–13.5)	17.79	(17.4–18.1)	5.23	(5.1–5.3)	7.94	(7.8–8.1)
55–59	26.54	(26.52–26.55)	31.64	(31.63–31.66)	17.67	(17.3–18)	20.93	(20.6–21.3)	10.96	(10.7–11.2)	14.95	(14.7–15.2)	4.51	(4.4–4.6)	7.01	(6.9–7.1)
65–74	18.76	(18.75–18.78)	22.88	(22.86–22.89)	13.06	(12.8–13.3)	15.15	(14.9–15.4)	8.04	(7.8–8.2)	11.12	(10.9–11.3)	3.87	(3.8–4)	5.74	(5.6–5.9)
75–84	11.87	(11.86–11.88)	14.52	(14.51–14.53)	7.33	(7.1–7.5)	8.35	(8.2–8.5)	5.2	(5–5.4)	6.68	(6.5–6.9)	2.75	(2.7–2.8)	4.14	(4–4.3)
85+	6.26	(6.26–6.26)	7.46	(7.46–7.46)	0.75	(0.7–0.8)^b^	0.87	(0.9–0.9)^b^	0.6	(0.6–0.6)^b^	0.72	(0.7–0.7)	0.48	(0.4–0.5)	0.59	(0.6–0.6)
Secondary or more																
45–54	37.37	(37.35–37.38)	42.67	(42.66–42.68)	19.78	(19.5–20.1)	22.28	(21.9–22.6)	8.84	(8.7–9)	11.93	(11.7–12.1)	3.03	(3–3.1)	5.07	(5–5.2)
55–59	28.21	(28.2–28.22)	33.17	(33.16–33.19)	16.66	(16.3–17)	18.6	(18.2–19)	7.43	(7.3–7.6)	9.85	(9.6–10.1)	2.6	(2.6–2.6)	4.37	(4.3–4.5)
65–74	19.93	(19.92–19.94)	24.12	(24.11–24.13)	12.19	(11.9–12.5)	13.52	(13.2–13.9)	5.43	(5.3–5.6)	7.31	(7.1–7.5)	2.19	(2.1–2.2)	3.67	(3.6–3.8)
75–84	12.58	(12.57–12.6)	15.47	(15.46–15.48)	6.81	(6.5–7.1)	7.53	(7.2–7.8)	3.37	(3.2–3.5)	4.25	(4.1–4.4)	1.46	(1.4–1.5)	2.57	(2.5–2.7)
85+	6.62	(6.62–6.63)	8.03	(8.02–8.03)	0.76	(0.7–0.8)^a^	0.82	(0.8–0.9)^a^	0.56	(0.5–0.6)^a^	0.53	(0.5–0.6)^a^	0.4	(0.4–0.4)^a^	0.43	(0.4–0.5)^a^

a Gender differences not significant.

b Educational differences not significant.

Regarding gender gaps, women spend more years in bad health (UHLE) than men for all measures and educational levels through all age groups, and these differences are significant when comparing 95% confidence intervals (years in good health results are available upon request). Regarding educational differences, those with low levels of education live more years in poor health. Both of these patterns apply at all ages with few exceptions at advanced ages 85+. On one hand, gender differences in UHLE at the oldest age group with higher education are not significantly different for all the health measures considered. On the other hand, UHLE differences between primary and secondary education are not significant at ages 85+ for chronic diseases among men and women, and for men with bad self-reported health.

### Gender and Educational Gaps in Unhealthy Life Expectancy for Each Health Measure Over the Lifespan

**FIGURE 2:**
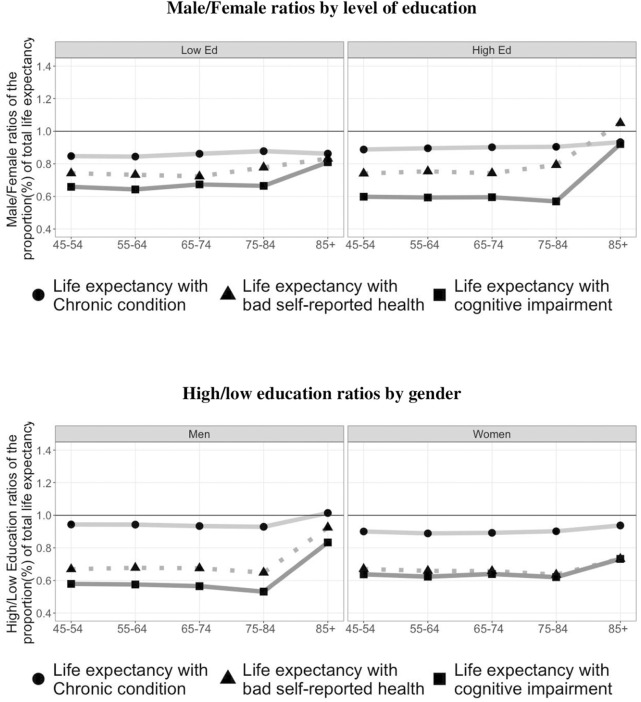
Ratios of the proportion of unhealthy life expectancy by gender and education. Individuals aged 45+ (Source: European Health Interview Survey (EHIS), Spanish Statistical Office (INE), Spain, 2019).

Figure 2 (top panel) shows gender ratios (male/female) of the expected years living in poor health for each health measure and level of education within 5 age groups (45–85+). In general, women in both educational groups spend a higher proportion of their lives in worse health than men through all ages. The only exception is life expectancy with bad self-reported health at ages 85+ for women with high education (although the gender gap was not significant). Thus, the gender paradox is confirmed for the two levels of education studied here. The most striking result is that cognitive impairment among individuals with high education shows the largest gender differences. At age 45 men spend 15% and 11% less years with chronic diseases than women with lower education and higher education, respectively. The corresponding ratio for years spent with cognitive impairment increases to 34% and 40% for men compared to women with lower and higher education, respectively. Overall, the gender gaps are similar for each health measure across age groups in both education levels. The observed reduction of the gender gap of UHLE at age 85+ with chronic disease and life with cognitive impairment draw on non-significant gender differences (Table 2).

Figure 2 (bottom panel) shows the education ratios (high/low) of the proportion of total life expectancy spent with health problems for men and women aged 45 to 85+. Among women, those with low levels of education spend a higher proportion of their lives in worse health than those with high levels of education for all three health measures and at all ages. The smallest education gap is observed for life spent with chronic disease. Differences are similar for the other two measures and across age groups, with a reduction at ages 85+. For men, we see a similar pattern than for women, although women show larger education rations than men, except for the years spent in cognitive impairments. Therefore, while individuals with high education at age 45 spend 6% (men) and 10% (women) less years with chronic diseases, the difference is greater than 30% for the rest of the measures analyzed. In particular, 45 years-old men with high levels of education are expected to live 42% less years with cognitive impairments than 45 years-old men with low education levels (the corresponding value for women is a difference of 36%).

## Discussion

Our findings show the persistence of marked gender differences in health across most ages 45+ and among those with low educational attainment. In particular, we found (1) longer life for women at all ages and at all levels of education, but with longer periods subject to health problems compared to men; (2) greater health inequalities by education level for women (except for the years lived with cognitive impairment); and (3) a health gradient associated with aging as well as across the health measures. Thus, we can confirm the gender paradox for all health measures, both educational levels, and across all ages. Regardless of age, and for both levels of education, women spend a higher proportion of their lives with chronic morbidity, worse self-rated health and cognitive impairment. Our results are consistent with previous evidence from a comparative study including more than a dozen countries from Europe, Asia and America that confirmed that at present women live longer than men and have more disabling chronic conditions while men have more lethal conditions [26]. However, this gender gap is greater among those with primary education with the exception of cognitive impairment. Those greater gender differences might reflect the lower employment rate and higher precariousness among Spanish women [27] among those with lower education, reflected in their greater involvement in care work across their life-course [28].

The socioeconomic differences observed here go in the expected direction. Those with low education levels spend more years in worse health than those with high education, a finding that holds across all ages. When comparing each health measure, the education ratios are lower when measuring the presence of chronic diseases. This might be attributable to the lower frequency of use of medical/health services by individuals with low education levels [29, 30], making them less likely to receive a diagnosis. Previous research has shown that women experience greater socioeconomic inequalities in morbidity measures [18], but gender inequalities across the social gradient are not often the focus of analysis. Our findings indicate that educational attainment is more beneficial for women than it is for men in terms of the proportion of life spent in good health, as the gains of increasing education are greater, with the exception of cognitive impairment. Our results suggest that both gender and education gaps in cognitive impairment reflect the better situation of high educated men compared to any other education and gender group, consistent with the lower prevalence observed, whether it responds to their better cognitive status or their better self-reported performance. Both morbidity and mortality patterns by gender and education might explain the observed relative advantage of high-educated men. These finding merits further research.

Here, for the first time in Spain, we have estimated life expectancy with cognitive impairment [31], a growing social concern given its associations with dementia and increased dependency among the expanding older population where it represents a major care burden. We found that lower educated men and women live more years with cognitive impairment despite leading shorter lives. These educational differences in life expectancy with cognitive impairment are consistent with previous research [32]. The marked differences found here, particularly among women, reflect the important role of education in delaying the onset of, and shortening, cognitive decline [33, 34]. This indicator is expected to improve as better-educated younger cohorts age. Indeed, the greater expansion of education among Spanish female cohorts may have a marked impact, even if age-specific prevalence is not reduced. Thus, we must be cautious when interpreting any improvements in future trends of life expectancy with cognitive impairment in Spain as they might reflect a change in the educational composition of the population rather than in adult cognitive ability as they age.

Our most relevant contribution is the comparison of gender and educational ratios of UHLE for different health measures and age groups among adults aged 45-plus. In sensitivity analysis, we performed the same analysis for years spent with activity limitation, measured from the Global Activity Limitation Index (GALI), and results were very similar to those from the more general measure of self-rated health (results upon request). As we have seen, the selected health measures used here present a gradient by age which, despite the cross-sectional nature of our study, reflects the aging (disabilty) process [35]—starting with chronic conditions and progressing with bad self-reported health, (disability problems, as sensitivity analysis revealed) and culminating in cognitive impairments–that only becomes substantially prevalent at older ages. Across middle and older ages, gender ratios are more strongly negative for women for measures that capture a more acute level of disability. Therefore, morbidity and mortality outcomes suggest that health interventions aimed at delaying the onset of disease would benefit men more than women, while practices that reduce health deterioration associated with disease would be more beneficial for women, as they are more likely to suffer the deteriorating sequelae of these diseases [36].

Three potential limitations that might affect our results should be mentioned. First, our study employs cross-sectional data and draws on different data sources. The latter leaves our results open to the possible risks associated with the distribution of deaths by level of education, which might be slightly different to the method used by INE [35], although observed differences were very small (less than 1%). Second, the subjective (self-reported) health measures used might be sensitive to potential bias caused by gender differences in reporting styles [7]. Moreover, self-reported measures might be sensitive to the level of development (social, economic and cultural). Partly, we have tried to address this potential shortcoming by combining self-reported and diagnosed health measures. Finally, the data sources used do not include the institutionalized older population and they are not included in our surveys. This might result in the underestimation of the real magnitude of some findings, particularly among those aged 85+.

Despite these limitations, this study highlights the important role played by education in subjective well-being and complements previous findings reported for Spain [14]. Specifically, it highlights that education has a more powerful impact on women’s health, particularly among older cohorts, with a higher proportion of low educated women. This result applies to all measures examined here but cognitive impairment where men benefit more from high education. Beyond the gender gap in life expectancy, education makes a difference in both objective and subjective health, which adds a new layer of vulnerability to that already suffered by the most vulnerable [37]. This study reaffirms the need to address educational and gender health inequalities in adult age to ensure a better quality of life in old age.

To conclude, our results support the argument that the changing distribution of education should improve the healthy life expectancy of the total population and reduce the gender gap in the number of years spent in poor health. However, while we await the arrival of more educated cohorts, reducing poor health for all, but especially women with primary or less education, should be a priority in contributing to active aging and delaying the disablement process. In line with our results, we recommend addressing age-specific prevalence of diseases by promoting effective health measures with a focus on their impact on the genders to enhance independent living for future generations of older men and women.

## References

[1] Crimmins EM Kim JK Solé-Auró A. Gender Differences in Health: Results from SHARE, ELSA, and HRS. Eur J Public Health (2011) 21(1):81–91. 10.1093/eurpub/ckq022 20237171 PMC3023013

[2] Deeg DJH Bath PA. Self-rated Health, Gender, and Mortality in Older Persons: Introduction to a Special Section. Gerontologist (2003) 43:369–71. 10.1093/geront/43.3.369 12810900

[3] Kulminski AM Culminskaya IV Ukraintseva SV Arbeev KG Land KC Yashin AI. Sex-specific Health Deterioration and Mortality: The Morbidity-Mortality Paradox over Age and Time. Exp Gerontol (2008) 43:1052–7. 10.1016/j.exger.2008.09.007 18835429 PMC2703431

[4] Di Lego V Di Giulio P Luy M. Gender Differences in Healthy and Unhealthy Life Expectancy. In: International Handbook of Health Expectancies. Berlin, Germany: Springer (2020). p. 151–72.

[5] Oksuzyan A Gumà J Doblhammer G. Sex Differences in Health and Survival. In: A Demographic Perspective on Gender, Family and Health in Europe. Cham: Springer (2018). p. 65–100.

[6] Oksuzyan A Brønnum-Hansen H Jeune B. Gender gap in Health Expectancy. Eur J Ageing (2010) 7(4):213–8. 10.1007/s10433-010-0170-4 28798630 PMC5547325

[7] Zacajova A Huzurbazar S Todd M. Gender and the Structure of Self-Rated Health across the Adult Life Span. Soc Sci Med (2017) 187:58–66. 10.1016/j.socscimed.2017.06.019 28654822 PMC5554534

[8] Crimmins EM Saito Y. Trends in Healthy Life Expectancy in the United States, 1970-1990: Gender, Racial, and Educational Differences. Soc Sci Med (2001) 52(11):1629–41. 10.1016/s0277-9536(00)00273-2 11327137

[9] Kc S Lentzner H. The Effect of Education on Adult Mortality and Disability: A Global Perspective. Vienna Yearb Popul Res (2010) 8:201–36. 10.1553/populationyearbook2010s201

[10] Cambois E Solé-Auró A Jeune B Brønnum-Hansen H Egidi V Jagger C Educational Differentials in Disability Vary across and within Welfare Regimes: a Comparison of 26 European Countries in 2009. J Epidemiol Community Health (2016) 70(4):331–8. 10.1136/jech-2015-205978 26546286

[11] Hu Y van Lenthe FJ Borsboom GJ Looman CWN Bopp M Burström N Trends in Socioeconomic Inequalities in Self-Assessed Health in 17 European Countries between 1990 and 2010. J Epidemiol Community Health (2016) 70(7):644–52. 10.1136/jech-2015-206780 26787202

[12] Reques L Giráldez-García C Miqueleiz E Belza MJ Regidor E. Educational Differences in Mortality and the Relative Importance of Different Causes of Death: A 7-year Follow-Up Study of Spanish Adults. J Epidemiol Community Health (2014) 68:1151–60. 10.1136/jech-2014-204186 25124190

[13] Permanyer I Spijker J Blanes A Renteria E. Longevity and Lifespan Variation by Educational Attainment in Spain: 1960–2015. Demography (2018) 55:2045–70. 10.1007/s13524-018-0718-z 30324395

[14] Solé-Auró A Martin U Domínguez-Rodríguez A. Educational Inequalities in Life and Healthy Life Expectancies Among the 50-plus in Spain. Int J Environ Res Public Health (2020) 17(10):3558. 10.3390/ijerph17103558 PMC7277913 32438706

[15] Blanes A Trias-Llimós S. Shorter Lives with Poor Health: The Toll on Spain’s Less Educated Population. Perspect Demogràfiques (2021) 24:1–4. 10.46710/ced.pd.eng.24

[16] Kulhánová I Bacigalupe A Eikemo TA Borrell C Regidor E Esnaola S Why Does Spain Have Smaller Inequalities in Mortality? an Exploration of Potential Explanations. Eur J Public Health (2014) 24:370–7. 10.1093/eurpub/cku006 24568755

[17] Regidor E Gutiérrez-Fisac JL Rodríguez C. Increased Socioeconomic Differences in Mortality in Eight Spanish Provinces. Soc Sci Med (1995) 41:801–7. 10.1016/0277-9536(94)00402-f 8571151

[18] Majer IM Nusselder W Mackenbach J Kunst A. Socioeconomic Inequalities in Life and Health Expectancies Around Official Retirement Age in 10 Western-European Countries. J Epidemiol Community Health (2011) 65:972–9. 10.1136/jech.2010.111492 21106546

[19] Instituto Nacional de Estadística (INE). Esperanza de Vida según sexo, edad y nivel educativo (2022). Available online: https://www.ine.es/jaxiT3/Tabla.htm?t=37663&L=0 http://www.ine.es/metodologia/t20/t2030306_niveduc.pdf (accessed March, 2022).

[20] Zueras P Rentería E. Trends in Disease-free Life Expectancy at Age 65 in Spain: Diverging Patterns by Sex, Region and Disease. PloS one (2020) 15(11):e0240923. 10.1371/journal.pone.0240923 33175856 PMC7657566

[21] Solé-Auró A Beltrán-Sánchez H Crimmins EM. Are Differences in Disability-free Life Expectancy by Gender, Race and Education Widening at Older Ages? 1986-2006. Popul Res Pol Rev (2015) 34(1):1–18. 10.1007/s11113-014-9337-6 PMC5906056 29681672

[22] Jagger C Van Oyen H Robine J. Health Expectancy Calculation by the Sullivan Method: A Practical Guide. Newcastle upon Tyne: Newcastle University Institute Ageing (2014). p. 1–40.

[23] Sullivan DF. A Single index of Mortality and Morbidity. Rockville, Md: Health Services and Mental Health Administration (1971). PMC1937122 5554262

[24] Jagger C Cox B Le Roy, S EHEMU team. Health Expectancy Calculation by the Sullivan Method: A Practical Guide. EHEMU Technical Report (2007).

[25] Villavicencio F Bergeron-Boucher MP Vaupel JW. Reply to Permanyer et al.: The uncertainty surrounding healthy life expectancy indicators. Proc Natl Acad Sci U S A (2021) 118(46): e2115544118. 10.1073/pnas.2115544118 34772817 PMC8609540

[26] Crimmins EM Shim H Zhang YS Kim JK. Differences between Men and Women in Mortality and the Health Dimensions of the Morbidity Process. Clin Chem (2019) 65(1):135–45. 10.1373/clinchem.2018.288332 30478135 PMC6345642

[27] Lozano M Solé-Auró A. Happiness and Life Expectancy by Main Occupational Position Among Older Workers: Who Will Live Longer and Happy. SSM Popul Health (2021) 13:100735. 10.1016/j.ssmph.2021.100735 33511266 PMC7815996

[28] Rentería E Scandurra S Souto G Patxot C. Intergenerational Money and Time Transfers by Gender in Spain: Who Are the Actual Dependents? Demogr Res (2016) 34(24):689–704. 10.4054/DemRes.2016.34.24

[29] Blomgren J Virta L. Socioeconomic Differences in Use of Public, Occupational and Private Health Care: A Register-Linkage Study of a Working-Age Population in Finland. PLoS One (2020) 15(4):e0231792. 10.1371/journal.pone.0231792 32298356 PMC7162494

[30] Palència L Espelt A Rodríguez-Sanz M Rocha KB Pasarín MI Borrell C. Trends in Social Class Inequalities in the Use of Health Care Services within the Spanish National Health System, 1993–2006. Eur J Health Econ (2013) 14:211–9. 10.1007/s10198-011-0362-7 22072321

[31] Matthews FE Jagger C Miller LL Brayne C Mrc CFAS. Education Differences in Life Expectancy with Cognitive Impairment. J Gerontol A Biol Sci Med Sci (2009) 64(1):125–31. 10.1093/gerona/gln003 19182231 PMC2691183

[32] Reuser M Willekens FJ Bonneux L. Higher Education Delays and Shortens Cognitive Impairment. A Multistate Life Table Analysis of the US Health and Retirement Study. Eur J Epidemiol (2011) 26(5):395–403. 10.1007/s10654-011-9553-x 21337033 PMC3109265

[33] Baumgart M Snyder HM Carrillo MC Fazio S Kim H Johns H. Summary of the Evidence on Modifiable Risk Factors for Cognitive Decline and Dementia: a Population-Based Perspective. Alzheimers Dement (2015) 11(6):718–26. 10.1016/j.jalz.2015.05.016 26045020

[34] Andrade FCD Corona LP de Oliveira Duarte YA. Educational Differences in Cognitive Life Expectancy Among Older Adults in Brazil. J Am Geriatr Soc (2019) 67(6):1218–25. 10.1111/jgs.15811 30715738

[35] Verbrugge LM Jette AM. The Disablement Process. Soc Sci Med (1994) 38(1):1–14. 10.1016/0277-9536(94)90294-1 8146699

[36] Instituto Nacional de Estadística (INE). Asignación de nivel educativo, relación con la actividad laboral y ocupación a ficheros de Movimiento Natural de la Población (MNP). Método de obtención y advertencias a usuarios (2020). Available online: https://www.ine.es/metodologia/t20/Nota_meto_MNP.pdf.

[37] Grundy E. Ageing and Vulnerable Elderly People: European Perspectives. Ageing Soc (2006) 26(1):105–34. 10.1017/S0144686X05004484

