# Corrigendum: Gender Gap in Unhealthy Life Expectancy: The Role of Education Among Adults Aged 45+

**DOI:** 10.3389/ijph.2022.1605411

**Published:** 2022-10-20

**Authors:** Aïda Solé-Auró, Pilar Zueras, Mariona Lozano, Elisenda Rentería

**Affiliations:** ^1^ Department of Political and Social Sciences, Pompeu Fabra University, Barcelona, Spain; ^2^ Center for Demographic Studies, Autonomous University of Barcelona, Barcelona, Spain; ^3^ Institute for Social and Economic Research, Colchester, United Kingdom

**Keywords:** population health, gender paradox, disablement process, education gradient, Spain

In the published article, the author order was incorrect regarding the final two authors Mariona Lozano and Elisenda Reneria. The corrected author list appears below.

Aïda Solé-Auró^1,^*, Pilar Zueras^2,3^, Mariona Lozano^2^ and Elisenda Rentería^2^

In the published article, there was an error in Table 2 as published. The columns for YEARS and CI are misaligned.

**TABLE 2:** Life expectancy and unhealthy life expectancy (in years) for both men and women and for low and high levels of education by ten-year age groups (Spain, 2019).

	Life Expectancy				Years with health problems (UHLE)											
					Chronic condition				Bad self-reported health				Cognitive impairment			
	Men		Women		Men		Women		Men		Women		Men		Women	
	Years	CI	Years	CI	Years	CI	Years	CI	Years	CI	Years	CI	Years	CI	Years	CI
Primary or less																
45–54	35.24	(35.22–35.26)	40.8	(40.79–40.82)	20.96	(20.5–21.4)	24.73	(24.3–25.2)	13.2	(12.9–13.5)	17.79	(17.4–18.1)	5.23	(5.1–5.3)	7.94	(7.8–8.1)
55–59	26.54	(26.52–26.55)	31.64	(31.63–31.66)	17.67	(17.3–18)	20.93	(20.6–21.3)	10.96	(10.7–11.2)	14.95	(14.7–15.2)	4.51	(4.4–4.6)	7.01	(6.9–7.1)
65–74	18.76	(18.75–18.78)	22.88	(22.86–22.89)	13.06	(12.8–13.3)	15.15	(14.9–15.4)	8.04	(7.8–8.2)	11.12	(10.9–11.3)	3.87	(3.8–4)	5.74	(5.6–5.9)
75–84	11.87	(11.86–11.88)	14.52	(14.51–14.53)	7.33	(7.1–7.5)	8.35	(8.2–8.5)	5.2	(5–5.4)	6.68	(6.5–6.9)	2.75	(2.7–2.8)	4.14	(4–4.3)
85+	6.26	(6.26–6.26)	7.46	(7.46–7.46)	0.75	(0.7–0.8)^b^	0.87	(0.9–0.9)^b^	0.6	(0.6–0.6)^b^	0.72	(0.7–0.7)	0.48	(0.4–0.5)	0.59	(0.6–0.6)
Secondary or more																
45–54	37.37	(37.35–37.38)	42.67	(42.66–42.68)	19.78	(19.5–20.1)	22.28	(21.9–22.6)	8.84	(8.7–9)	11.93	(11.7–12.1)	3.03	(3–3.1)	5.07	(5–5.2)
55–59	28.21	(28.2–28.22)	33.17	(33.16–33.19)	16.66	(16.3–17)	18.6	(18.2–19)	7.43	(7.3–7.6)	9.85	(9.6–10.1)	2.6	(2.6–2.6)	4.37	(4.3–4.5)
65–74	19.93	(19.92–19.94)	24.12	(24.11–24.13)	12.19	(11.9–12.5)	13.52	(13.2–13.9)	5.43	(5.3–5.6)	7.31	(7.1–7.5)	2.19	(2.1–2.2)	3.67	(3.6–3.8)
75–84	12.58	(12.57–12.6)	15.47	(15.46–15.48)	6.81	(6.5–7.1)	7.53	(7.2–7.8)	3.37	(3.2–3.5)	4.25	(4.1–4.4)	1.46	(1.4–1.5)	2.57	(2.5–2.7)
85+	6.62	(6.62–6.63)	8.03	(8.02–8.03)	0.76	(0.7–0.8)^a^	0.82	(0.8–0.9)^a^	0.56	(0.5–0.6)^a^	0.53	(0.5–0.6)^a^	0.4	(0.4–0.4)^a^	0.43	(0.4–0.5)^a^

a Gender differences not significant.

b Educational differences not significant.

The corrected Table 2 and its caption appear below.

The authors apologize for this error and state that this does not change the scientific conclusions of the article in any way. The original article has been updated.

